# Recognition capability of one’s own skilled movement is dissociated from acquisition of motor skill memory

**DOI:** 10.1038/s41598-021-96381-w

**Published:** 2021-08-18

**Authors:** Nobuaki Mizuguchi, Shohei Tsuchimoto, Hirofumi Fujii, Kouki Kato, Tomoyuki Nagami, Kazuyuki Kanosue

**Affiliations:** 1 Research Organization of Science and Technology, Ritsumeikan University, 1-1-1 Noji-Higashi, Kusatsu, Shiga 525-8577 Japan; 2 Faculty of Sport Sciences, Waseda University, 2-579-15 Mikajima, Tokorozawa, Saitama 359-1192 Japan; 3 Division of System Neuroscience, National Institute for Physiological Sciences, Aichi, 444-8585 Japan; 4 Physical Education Center, Nanzan University, 18 Yamazato, Aichi, 466-8673 Japan; 5 College of Liberal Arts and Sciences, Kitasato University, 1-15-1 Kitazato, Minami-ku, Sagamihara, Kanagawa 252-0373 Japan

**Keywords:** Motor control, Learning and memory

## Abstract

When we have rehearsed a movement using an object, we can reproduce the movement without holding the object. However, the reproduced movement sometimes differs from the movement holding a real object, likely because movement recognition is inaccurate. In the present study, we tested whether the recognition capability was dissociated from the acquisition of motor skill memory. Twelve novices were asked to rotate two balls with their right hand as quickly as possible; they practiced the task for 29 days. To evaluate recognition capability, we calculated the difference in coordination pattern of all five digits between the ball-rotation movement and the reproduced movement without holding balls. The recognition capability did not change within the first day, but improved after one week of practice. On the other hand, performance of the ball rotation significantly improved within the first day. Since improvement of performance is likely associated with acquisition of motor skill memory, we suggest that recognition capability, which reflects the capability to cognitively access motor skill memory, was dissociated from the acquisition of motor skill memory. Therefore, recognition of one’s own skilled movement would rely on a hierarchical structure of acquisition of motor skill memory and cognitive access to that memory.

## Introduction

We can recognize the result of our own movement via somatosensory feedback as well as visual feedback. However, without visual feedback, the recognized movement can sometimes be dissociated from the kinematics of the actual movement, especially in complex motor skills. For example, a skier might believe that they are a moderate skier, only to be disappointed when they view a video recording of their poor skiing performance. The movement of a hand holding a real object may differ from the same movement reproduced using memory without holding the object. The reproduced movement is likely based on internally recognized movement^1^. Therefore, dissociation of the reproduced movement from the movement using an object originates from inaccurate recognition of the movement. Thus, the reduction of such subjective–objective movement dissociation is one important issue for effective motor skill learning.

Since motor skill is important for our daily activities including sports, music, and art, many previous studies have investigated neural mechanisms of motor control and skill learning^2^. In these studies, sequential tasks such as sequential finger tapping were often used as experimental models of motor skill tasks^3–5^. The accumulating evidence indicated that cognitive control or strategy is utilized in the early phase of motor skill learning. Then, the contribution of cognitive control or strategy decreases as motor skill memory is acquired in the cortico-subcortical regions^2^. Motor skill learning is important not only for improving motor performance but also for accurately recognizing one’s own movement, because the brain detects deviations of actual movement from intended movement by estimating the mismatch between predicted and actual outcomes (i.e., prediction error) utilizing the internal forward model^6^. The prediction error is also used for understanding the actions of others^7–9^. A neuroimaging study of action observation demonstrated that successfully adding a motor skill to one’s own motor repertoire is associated with the activation level of the action observation network (i.e., the frontoparietal network) rather than with visual familiarity^10^. Thus, acquisition of motor skill memory is an important factor for accurately estimating one’s own movements as well as the movements of others.

However, a recent visuomotor adaptation study suggests that recall of somatic motor memory as evaluated by the reproduced performance of a task could fail due to inaccessibility of the memory of the newly learned movement even though the somatic motor memory was surely acquired^11^. Thus, acquisition of motor memory and cognitive access to motor memory are likely dissociated. Indeed, it is well known that sometimes, when we cannot retrieve something (e.g., a name), if sensory retrieval cues or related information are provided we are then able to retrieve it^12^. In this case, the memory was stored in the brain but the brain failed to access the memory. Thus, the acquisition of motor memory might not be enough to enable accurate recognition of one’s own movements via somatosensory feedback. It is well known that the neural mechanism of sensorimotor adaptation is different from that of motor skill learning^13^. That is, sensorimotor adaptation tasks update the internal forward model (i.e., motor-to-sensory mapping) based on errors generated by perturbation or environmental changes, but motor skill learning stores motor skill memory and creates an internal model in the brain, usually without any perturbation^13^. Therefore, it remains unclear whether the acquisition of motor skill memory and the capability of cognitive access to motor skill memory are dissociated.

Many sports movements require smooth coordination of many body parts (i.e., several muscles working together) by optimizing joint displacement and contraction timing. In this study, we considered a complex motor skill to be a movement with spatiotemporal coordination of several muscles and joints. Although movement complexity (e.g., number of muscles and time constraints) is likely the crucial determinant of the difficulty of cognitive access to motor skill memory, motor skill tasks which were utilized in previous studies were relatively simple as compared with complex sport skills^4,5^. Therefore, it would be difficult to detect the dissociation of the acquisition of motor skill memory from the capability of access to motor skill memory. Indeed, a previous study argued that the basis of simple motor skills could not always be generalized to more complex motor skills^14^. Thus, to investigate the dissociation of the acquisition of motor skill memory from the capability of cognitive access to the motor skill memory, it is necessary to analyze a complex motor skill task.

Previous studies demonstrated that motor skill learning relies on several processes such as use-dependent plasticity, reinforcement, and error-based learning^15,16^. In addition, the contribution of each of these processes varies with learning phase (i.e., early or late phase)^17^. Therefore, some previous neuroimaging studies adopted a several-weeks practice schedule to clarify time-dependent acquisition of memory representation of motor skill components^18–20^. The recognition capability for one’s own skilled movement would be one of the motor skill components. Thus, the learning curve of the recognition capability might differ from that of the acquisition of motor skill memory as reflected by the performance level of the motor skill.

In the present study, we used a ball-rotation movement as an experimental model to analyze a complex motor skill. This movement requires smooth coordination of all digits by optimizing their displacement and timing, with little cognitive demand^21^. Thus, it can be difficult to accurately discern the coordination pattern of all digits, especially in the early phase of motor learning. A previous study used the reproduction task to evaluate the accessibility of motor memory^11^. Thus, we also conducted a reproduction task without holding balls (REP) (i.e., pantomime) to estimate the spatio-temporal accuracy of the internally recognized movement obtained by referencing the motor skill memory. Then, we calculated the difference in digit coordination patterns between the ball-rotation task (BRT) and the REP (i.e., reproduction capability). If participants were able to access their motor skill memory and accurately recognize their own movement, the difference in digit coordination pattern between the two tasks should be small. In the present study, the participants practiced the ball-rotation movement for 29 days including five evaluation days conducted every week (i.e., day 1, day 8, day 15, day 22, and day 29). We hypothesized that both the acquisition of motor skill memory as evaluated by the performance of the task, and the recognition capability of skilled movement as evaluated by the difference in digit coordination patterns would improve along with motor skill learning, but their learning curves could differ because the acquisition of these two capabilities is likely dissociated. In addition, in the later phase of motor learning, we predicted that both capabilities would plateau.

## Results

**Figure 1:**
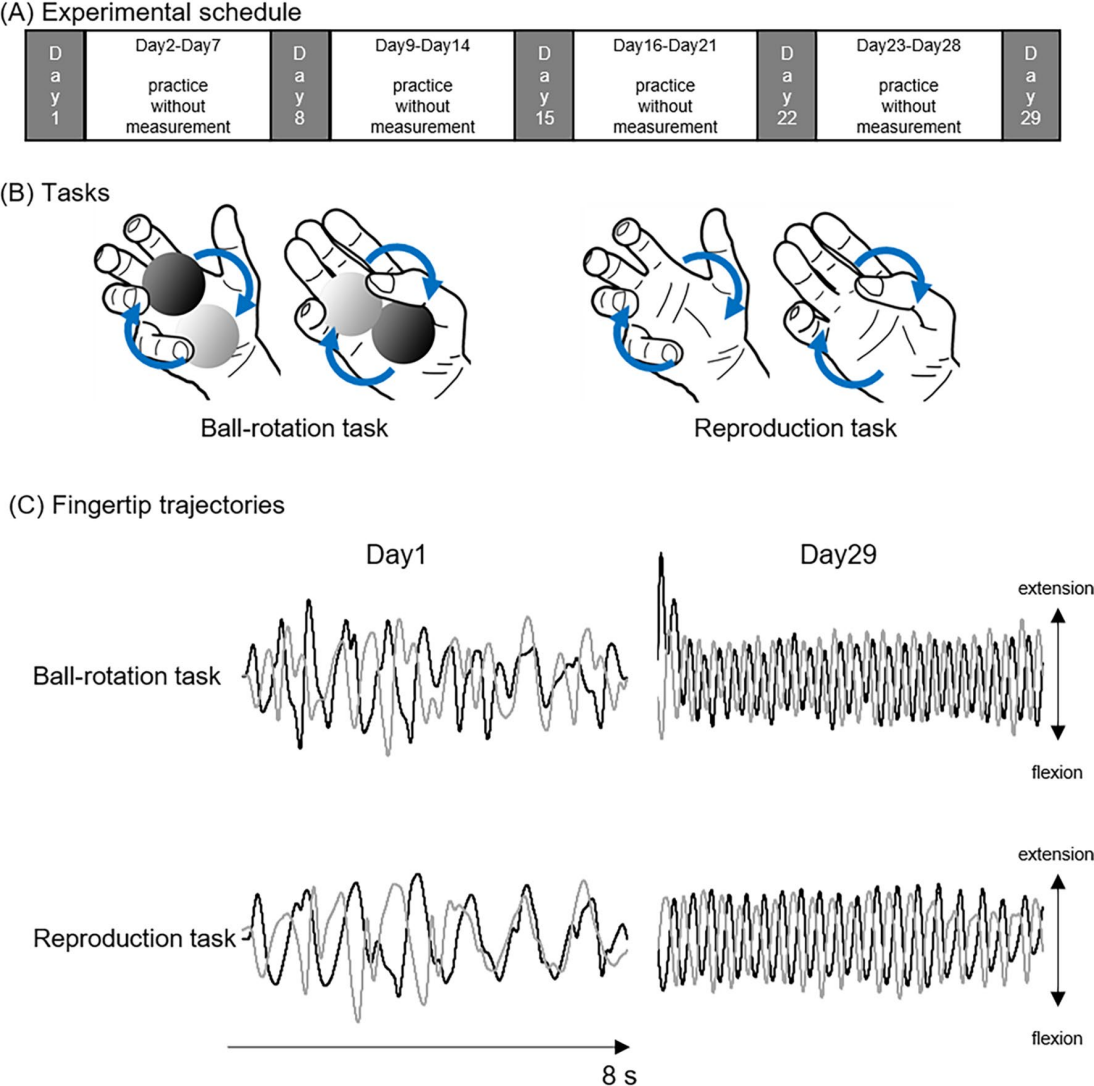
(**A**) Experimental schedule. Twelve naïve participants practiced a ball-rotation task for 29 days and completed 5 measurement days on day 1, day 8, day 15, day 22, and day 29. On each experimental day, a ball rotation task and a reproduction task were conducted. (**B**) Motor tasks: the ball-rotation task and the reproduction task. In the ball-rotation task, the participants were asked to rotate two balls around each other in a clockwise direction as quickly as possible. In the reproduction task, the participants were asked to reproduce the movement of the ball rotation as accurately as possible. (**C**) Typical waveforms of thumb and index finger tips in an 8-s trial from one participant on day 1 and day 29. Black lines indicate thumb and grey lines indicate index finger. Waveforms of the other three digits (middle, ring, and little fingers) are not shown in this figure because of complexity.

We trained 12 naïve participants to perform the ball rotation for 29 days (Fig. 1A). The participants completed five measurement days, performing the BRT and the REP on each measurement day. In the BRT, the participants were asked to rotate two balls around each other in a clockwise direction as quickly as possible (Fig. 1B). In the REP, the participants were asked to reproduce the digit movements of the ball rotation as accurately as possible (Fig. 1B). The time spent for one cycle (i.e., one rotation) and the difference in digit coordination pattern between the BRT and the REP were calculated using the fingertip trajectories of all digits as indices of the performance level of the ball-rotation and of the reproduction capability, respectively (Fig. 1C).

### Performance level of the motor task

**Figure 2:**
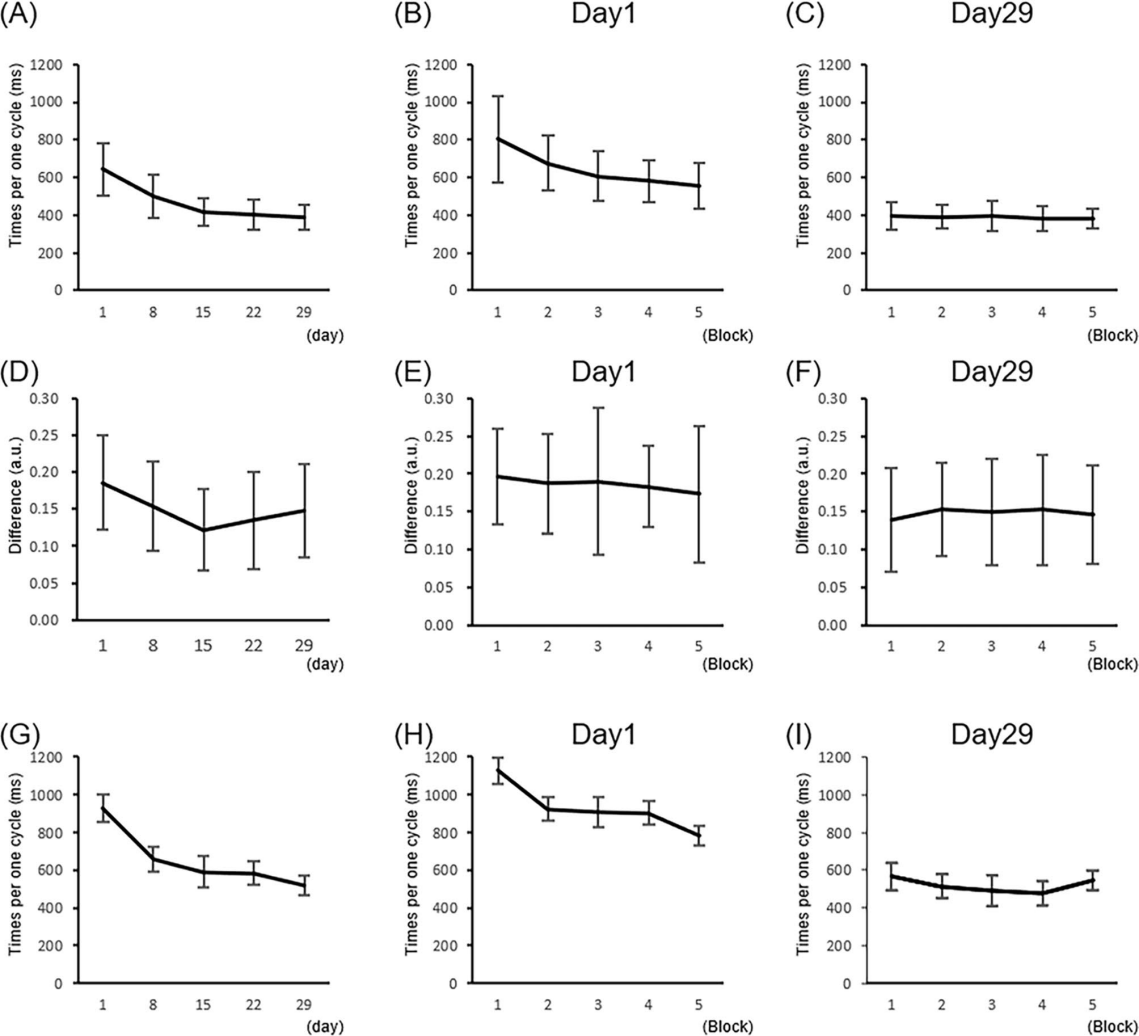
(**A**) Time spent for one cycle in the ball-rotation task across measurement days (day 1 vs. day 8: *p* = 5.8 × 10^–4^, d = 1.16; day 8 vs. day 15: *p* = 0.002, d = 0.84). (**B**) Time spent for one cycle in the ball-rotation task on day 1. Significant improvement was observed in only one block practice (block 1 vs. block 2: *p* = 0.0017, d = 0.67). (**C**) Time spent for one cycle in the ball-rotation task on day 29. Significant improvement was not observed (p > 0.0125). (**D**) Difference in digit coordination pattern between the ball-rotation and the reproduction tasks across measurement days. The difference significantly decreased from day 1 to day 8 (*p* = 0.009, d = 0.52). (**E**) Difference in digit coordination pattern between the ball-rotation and the reproduction tasks on day 1. The difference did not change within day 1 (*p* > 0.0125). (**F**) Difference in digit coordination pattern between the ball-rotation and the reproduction tasks on day 29. The difference did not change within day 29 (*p* > 0.0125). (**G**) Time spent for one cycle in the reproduction task across measurement days (day 1 vs. day 8: *p* = 5.5 × 10^–4^, d = 1.47). (**H**) Time spent for one cycle of the reproduction task on day 1. Significant improvement was observed within day (block 1 vs. block 5: *p* = 0.0016, d = 1.32). (**I**) Time spent for one cycle in the reproduction task on day 29. Significant improvement was not observed (*p* > 0.0125). Error bars indicate 1 standard deviation.

The time spent for one cycle in the BRT improved across measurement days (*F* (1.9, 21.3) = 39.71, *p* = 7.6 × 10^–9^ partial η^2^ = 0.783) (Fig. 2A). Post-hoc tests showed that the improvement was significant from day 1 to day 15 (day 1 vs. day 8: *t* (11) = 4.7, *p* = 5.8 × 10^–4^, d = 1.16; day 8 vs. day 15: *t* (11) = 3.9, *p* = 0.002, d = 0.84). After day 15, the time spent for one cycle did not improve significantly (day 15 vs. day 22: *t* (11) = 1.6, *p* = 0.12, d = 0.19; day 22 vs. day 29: *t* (11) = 1.4, *p* = 0.18, d = 0.18), indicating that motor skill performance had plateaued.

The time spent for one cycle in the BRT was significantly improved within day 1 (i.e., across 5 blocks) (*F* (1.4, 15.7) = 28.01, *p* = 2.2 × 10^–5^, partial η^2^ = 0.718) (Fig. 2B). Post-hoc tests showed that significant improvement was observed in only one block (i.e., 10 trials) of practice (block 1 vs. block 2: *t* (11) = 4.1, *p* = 0.0017, d = 0.67). On the final measurement day, the time spent for one cycle was not changed within day (Fig. 2C) (*F* (1.8, 19.8) = 3.19, *p* = 0.07, partial η^2^ = 0.225). Therefore, we also confirmed that motor skill performance had plateaued across blocks by day 29.

### Reproduction capability

The difference in digit coordination pattern between the two tasks became smaller across measurement days (*F* (2.7, 30.1) = 8.84, *p* = 3.3 × 10^–4^, partial η^2^ = 0.445) (Fig. 2D). That is, the difference significantly decreased from day 1 to day 8 (*t* (11) = 3.2, *p* = 0.009, d = 0.52). In addition, the difference marginally decreased from day 8 to day 15 (*t* (11) = 2.5, *p* = 0.028, d = 0.55). The difference did not alter from day 15 to day 29 (day 15 vs. day 22: *t* (11) = 1.0, *p* = 0.33, d = 0.22; day 22 vs. day 29: *t* (11) = 2.1, *p* = 0.06, d = 0.19). We also checked to ensure that day 15 was not significantly different from day 29 (*t* (11) = 1.9, *p* = 0.08, d = 0.44). Therefore, this trend was similar to that of the time spent for one cycle, indicating that the reproduction capability evaluated by the difference in digit coordination pattern improved, then plateaued. On the other hand, the difference in digit coordination pattern did not change within day 1 (i.e., within 5 blocks) (*F* (4,44) = 0.41, *p* = 0.80, partial η^2^ = 0.036) (Fig. 2E). This result implies that the recognition capability or the capability of cognitive access to the motor skill memory was not improved over the five experimental blocks of the first day. We also performed a direct comparison between normalized values of improvements in the time spent for one cycle and the difference in digit coordination pattern on day 1. We observed a greater improvement in the time spent for one cycle as compared to the difference in digit coordination pattern (*z* = 2.05, *p* = 0.04, d = 1.07). Therefore, our results indicate that the capability of cognitive access to the motor skill memory is dissociated from the acquisition of motor skill memory. The difference in digit coordination pattern did not change within day 5 (Fig. 2F) (*F* (4, 44) = 0.63, *p* = 0.65, partial η^2^ = 0.05).

The time spent for one cycle in the REP across measurement days improved in a similar way to the BRT (*F* (1.9, 21.1) = 22.52, *p* = 6.9 × 10^–6^, partial η^2^ = 0.672) (Fig. 2G). Post-hoc tests showed that the improvement was significant from day 1 to day 8 (*t* (11) = 4.8, *p* = 5.5 × 10^–4^, d = 1.47). In addition, the time spent for one cycle in the REP became significantly faster within day 1 (*F* (4, 44) = 5.82, *p* = 0.001, partial η^2^ = 0.346; block 1 vs. block 5: *t* (11) = 4.2, *p* = 0.0016, d = 1.32) (Fig. 2H), even though the accuracy of the digit coordination pattern did not change, as mentioned above. Therefore, changes in REP movement speed were not accompanied by spatio-temporal accuracy in the early phase of motor learning. On day 29, repeated measures analysis of variance (rmANOVA) showed significance (*F* (2.0, 22.0) = 3.75, *p* = 0.04, partial η^2^ = 0.254) (Fig. 2I). However, the significant improvement was not observed in post-hoc tests (*p* > 0.0125), indicating that the REP movement speed had also plateaued.

The time spent for one cycle in the REP was consistently greater than the time spent for one cycle in the BRT even on day 29. The time difference between the BRT and the REP did not correlate with the difference in digit coordination pattern between the BRT and the REP on day 1 (*p* = 0.34, r = 0.30) or on day 29 (*p* = 0.32, r = 0.31). Therefore, in the REP, the spatio-temporal accuracy of the digit coordination pattern was not associated with the speed of the whole movement.

**Figure 3:**
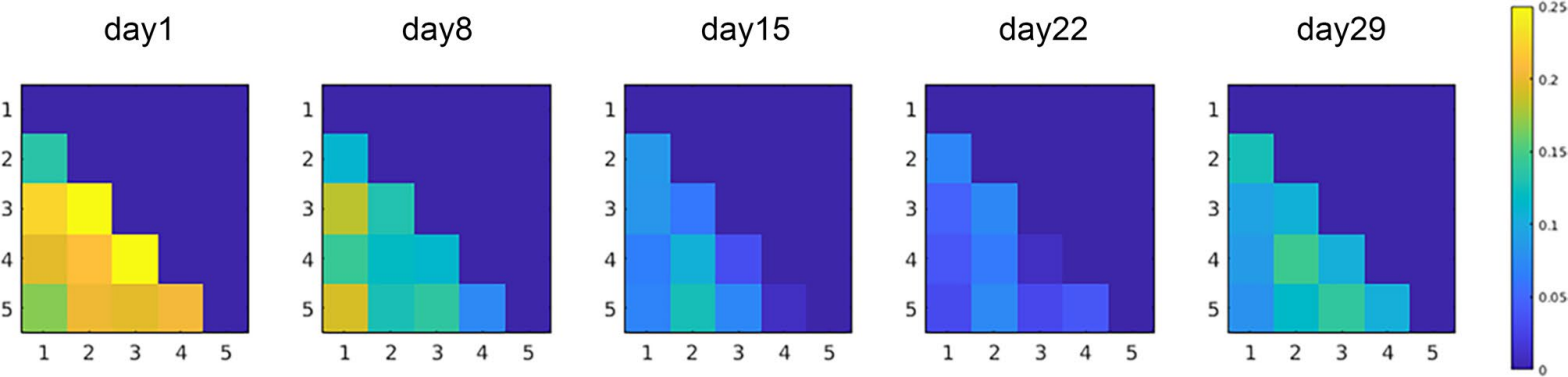
Difference in digit coordination patterns; combination of digits used in the reproduction task vs. the typical coordination pattern of the ball-rotation movement across measurement days. 1 = thumb, 2 = index finger, 3 = middle finger, 4 = ring finger, 5 = little finger. Zero indicates a coordination pattern that is identical to the typical coordination pattern of the ball-rotation movement.

To check whether the digit coordination pattern during the REP changed in all five digits or in specific digit combination patterns, the differences in 10 combinations (i.e., thumb-index finger, thumb-middle finger, ring-little fingers, etc.) from the typical movement found during the BRT is shown in Fig. 3. The differences in digit coordination pattern between the BRT and REP were decreased irrespective of the combination of digits. Therefore, decreased difference in digit coordination pattern would stem from the improvement of the reproduction for coordination pattern of all five digits rather than changes in a specific digit combination.

### Relationship between performance level and reproduction capability

**Figure 4:**
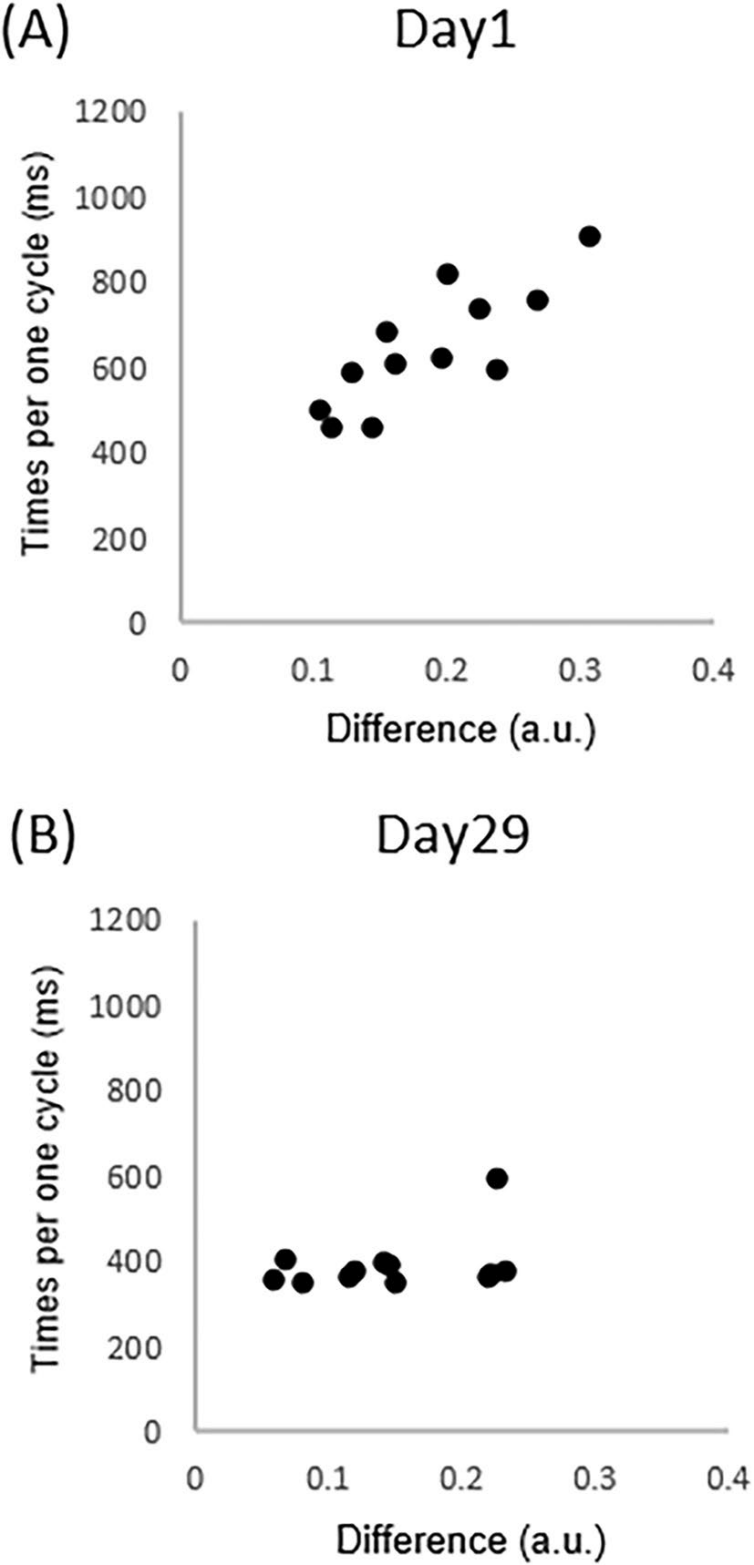
(**A**) Relationship between the difference in digit coordination pattern and the time spent for one cycle on day 1 (*p* = 0.0013, r = 0.81). (**B**) Relationship between the difference in digit coordination pattern and the time spent for one cycle on day 29 (*p* = 0.37, r = 0.24).

To investigate the relationship between performance level of the ball rotation and recognition capability, we calculated the correlation coefficient of time spent for one BRT cycle and the difference in digit coordination pattern. We found positive correlation on day 1 (*p* = 0.0013, r = 0.81) (Fig. 4A). This indicated that the participants with higher recognition capability (i.e., smaller difference in digit coordination pattern between the BRT and the REP) were able to perform the BRT faster in the initial phase of motor learning. However, the significance of this correlation was diminished on day 8 (*p* = 0.66, r = 0.14). Therefore, the performance level of the BRT in the middle phase of motor leaning is associated with other skill components rather than with recognition capability. In addition, a significant correlation was not observed on day 29 (*p* = 0.37, r = 0.24) (Fig. 4B), indicating that the recognition capability did not determine the plateaued level of the BRT. That is, the plateaued level of the BRT was limited by other factors.

We also checked whether the learning rate of the time spent for one cycle in the BRT (i.e., the difference between block 1 and block 5) and the recognition capability were associated on day 1. These were also correlated significantly (*p* = 0.012, r = 0.69), indicating that the participants with the larger difference improved more. We interpreted this to mean that the recognition capability would determine the initial performance level; thus, the learning rate would be small because the learning rate and the initial performance were also strongly correlated (*p* = 0.0062, r = 0.74).

## Discussion

Since recognition of simple, easy movements via somatosensory feedback is accurate, the recognized movement or the reproduced movement without holding the object is rarely dissociated from the actual simple movement, at least in healthy participants. In the present study, using a complex ball-rotation task, we tested whether the reproduction capability is dissociated from the performance level of the motor skill. We evaluated the difference in digit coordination pattern between the ball rotation and the reproduced movements as an index of the reproduction capability. In addition, the time spent for one cycle of ball rotation was calculated as an index of the performance level of the motor skill. Performance improved within the first day (Fig. 2B). On the other hand, the reproduction capability was not changed on the first day (Fig. 2E), but improved with one week of practice (Fig. 2D). It is possible that the participants recognized their own movement accurately but could not perform the REP. However, a previous study suggested that the reproduced movement of tool-use action without holding the tool is likely based on internally recognized movement^1^. Thus, the reproduction capability would reflect the participant’s ability to recognize their own skilled movement. Since performance level is likely associated with the acquisition of motor skill memory^2^, our results suggest that learning the recognition capability of a complex skilled movement progresses more slowly than acquiring the motor skill memory. The recognition capability is likely reflected in the capability of cognitive access to the motor skill memory^11^. If this view is correct, the acquisition of motor skill memory is dissociated from the capability of cognitive access to motor skill memory. In other words, acquisition of motor skill memory alone would not be enough for accurate recognition of movements. Therefore, recognition of the actions required to produce skilled movement would rely on a hierarchical process: (1) acquisition of motor skill memory or internal forward model, and (2) the ability to cognitively access that memory or model.

The difference in digit coordination pattern between two tasks decreased from day 1 to day 15. Since both the acquisition of motor skill memory and the capability of cognitive access to motor skill memory progress gradually by motor skill learning, spurious correlation of these two indices might be observed when participants exhibiting a wider range of performance level or phase were investigated (e.g., novice vs. expert or early phase vs. late phase). That is, subjective–objective dissociation would only be detectable in the early phase of learning complex motor skills because the capability of cognitive access to motor skill memory is likely undeveloped in this phase. The difference in digit coordination pattern plateaued around day 15 but the difference did not reach zero (i.e., movements with and without balls were not identical). This could be caused by the influence of peripheral factors such as external stimulation from the balls (i.e., mechanical constraints). Therefore, we believe that the plateaued behaviors in the REP signify a well-trained state rather than an incomplete state of training.

Interestingly, the reproduction capability in the initial phase was correlated with the initial BRT performance level (Fig. 4A). This implies that participants with accurate recognition of their skilled movement can better perform a novel complex motor skill. The strength of digits might be a confounder because motor skills as well as strength of digits affect the speed of ball rotation. The individual differences in the strength of digits, however, would not be associated with the initial performance level because the time spent for one cycle plateaued around 400 ms in most participants, indicating that the strength of digits was similar across participants, at least in the later phase of motor learning. That is, the strength of digits would limit the maximum rotation speed for every participant because the metal balls we used in the present study were relatively heavy. Unfortunately, we did not measure the strength of digits. However, not only in the later phase but also in the initial phase of learning the strength of digits could have been similar across participants because the participants completed the same amount of motor practice. Therefore, we believe that the recognition capability of skilled movement, rather than muscle strength, determines the initial performance level of unacquired complex motor skills. It is commonly observed that initial performance levels of motor skills vary across participants. Indeed, a recent study suggested that the experience of playing ball sports is associated with the acquisition of novel ball-related skills^22^. It was suggested that prediction of action outcomes likely determines the amount of motor learning^23^. Thus, participants with higher recognition capability would have better performance because they have a better capability to predict the outcome of novel actions. Such individual difference in motor performance is also considered to be the result of interaction between genetic and environmental factors involving motor experiences^24^. Thus, it would be interesting to investigate the relationship between the recognition capability and genetic or environmental factors. In addition, clarification is required to elucidate whether the recognition capability is determined depending on each motor skill (e.g., finger movements, whole body movements, etc.) or is more general among various motor skills.

On the first experimental day, the time spent for one cycle in the REP and the difference in digit coordination pattern show different temporal changes. That is, the speed of the reproduced movement became faster over the course of the day without any improvement of the spatio-temporal accuracy of the digit coordination pattern. It is possible that the movement speed in the REP was modulated explicitly in some participants. If speed of the reproduced movements increased in parallel with acquisition of motor skill memory, not only the movement speed but also the accuracy of the movement would have improved because the participants were instructed to reproduce the ball rotation movements with spatio-temporal accuracy rather than with total temporal accuracy (i.e., movement speed). However, the difference in digit coordination pattern did not change on the first day, as mentioned above. Therefore, it would be difficult to improve the accuracy of the spatio-temporal digit coordination pattern by explicit or cognitive strategy in the early phase of motor learning. Previous studies on action observation demonstrated that acquisition of motor skills is important for understanding complex movements, rather than visual familiarity or higher-order decision-making strategies^10,25^. That is, action observation automatically activates sensorimotor regions (i.e., the mirror neuron system)^26^. In addition, action prediction after observing the action implicitly affects the observer’s actions (i.e., motor contagions)^9^. This indicates that access to the motor skill memory is processed implicitly since implicit action prediction includes the process of accessing the motor skill memory. Thus, the ability to recognize one’s own skilled movement could partially rely on an implicit process and is likely not associated with higher-order decision-making strategies or knowledge. However, we were not able to exclude the possibility that knowledge (e.g., how to move the five digits) affected the performance of the REP. Therefore, it would be useful to test whether observing the actions during BRT learning improves REP performance. In addition, a neuroimaging study will be needed to clarify which system is associated with higher-order decision-making strategies.

Motor imagery is the mental process which recognizes the predicted sensory feedback of a movement without any actual sensory feedback^27^. Since motor imagery and motor execution share common neural substrates^28,29^, motor imagery training is widely utilized in sports, rehabilitation, and brain machine interfaces^30,31^. An elegant psychophysical study has recently suggested that the brain’s internal forward models predict the sensory consequences of imagined movements using efference copy as they do for overt movements^27^. Thus, the quality of motor imagery would depend on the internal model or the motor skill memory which is acquired in the brain with motor learning. However, a neuroimaging study suggested that subjective vividness of motor imagery was not always correlated with the actual motor performance level or brain activity in the motor related area during motor imagery^32^. Therefore, the present result that acquisition of motor skill memory is dissociated from the capability of cognitive access to the motor skill memory is consistent with the previous studies on motor imagery.

In the context of motor imagery training, the dissociation of speed and accuracy in the REP provides useful evidence. Previous studies demonstrated that speed of imagined movement evaluated by mental chronometry was slow as compared with that of actual movement when the participants imagined difficult movement^33^. In the present study, movement speed in the REP was consistently slower than that in the BRT. However, the changes in movement speed were not necessarily accompanied by the spatio-temporal accuracy of the reproduced movement. Therefore, motor imagery capability evaluated with mental chronometry would not be guaranteed to reflect the kinematical accuracy of motor imagery if it is assumed that the recognition capability acts commonly for both movement reproduction and motor imagery. The effect of motor imagery training would depend on motor imagery capability^34^. We considered that evaluating the kinematical aspect of motor imagery capability is important because the brain’s internal forward models predict the sensory consequences of imagined movements^27^. In other words, motor imagery with kinematically inaccurate movement cannot provide learnable somatosensory prediction and/or prediction error. This is consistent with a previous study demonstrating that motor imagery training did not improve for a totally novel movement^35^.

In the present study, there are several limitations. First, we only analyzed the trajectory of the fingertips of five digits. Therefore, we are not able to speculate about the joint coordination pattern involving the distal interphalangeal, proximal interphalangeal, and metacarpal phalangeal joints since these joints rarely appeared in the video (i.e., they were hidden by other digits or the balls). However, the peak timing of fingertip trajectory would reflect the sum of these joint movements. Therefore, we think that although analysis of digit coordination patterns in the present study is not perfect, it is sufficient to detect differences in the whole digit coordination pattern. Second, although the ball-rotation task is an action that uses an object, it is not an action that uses a tool. Tools are objects that we use as body extensions to interact with the environment^36^. Therefore, further studies are needed to test whether our results from the ball-rotation task can be generalized to a tool-use action, such as using a hammer. Furthermore, investigating various motor skills with different levels of complexity would be useful to enable us to define “complexity”. That is, even though the complexity of an action depends on action goal, context, and learning, one factor of complexity is a lack of accurate recognition of one’s own movements. Third, we did not conduct measurements from day 2 to day 7 even though significant changes in recognition capability should occur in these days. Therefore, details of the reproduction capability learning curve and the motor skill performance level remain unclear. Previous studies suggested that sleep is an important factor for memory consolidation^3^. Thus, the recognition capability might be improved, especially on day 2, with sleep during the previous night. In future study, it will be useful to clarify the time-dependent improvement in more detail. Fourth, our sample size was relatively small even though the effect size of the difference in digit coordination pattern within day 1 (Fig. 2E) was very low. In addition, it is possible that gender differences could exist; we studied only male participants^37^. Thus, a replication study without gender bias would be useful to establish generalized understanding.

## Conclusion

To investigate whether acquisition of motor skill memory is dissociated from the recognition capability for skilled movement, we utilized ball rotation as a model of complex tool-use skill. The results showed that the learning curve of the reproduction capability as assessed with the difference in digit coordination pattern between the ball rotation and the reproduced movement without balls differed from that of the performance level of ball rotation. Therefore, the recognition capability for skilled movement is apparently dissociated from the acquisition of motor skill memory. In addition, our results suggest that the recognition capability for skilled movement is a motor skill component, and thus determines the initial performance of complex motor skills with tools.

## Methods

### Participants

Twelve right-handed male novices (age: range 20–24 years, mean 22 ± 1) were enrolled in this study. All participants received a detailed explanation of the experimental procedures before the experiment and gave informed consent. The study was approved by the Human Research Ethics Committee of Waseda University (No. 2018-144). All experiments were carried out according to the principles and guidelines of the Declaration of Helsinki (1975).

### Motor tasks

The BRT is the main motor task. In this task, the participants continuously rotated two metal balls around each other clockwise (diameter 35 mm; 174.4 g each) in the palm of the right hand as many times as possible over each trial period of 8 s (Fig. 1B). The participants could not see their hand during either task (BRT or REP) because a partition was placed between their right hand and their face. On measurement days (see below), the participants also performed the REP. In the REP, the participants were asked to reproduce the digit movements of the BRT without holding the balls (i.e., pantomime) (Fig. 1B) as accurately as possible; movement speed was not emphasized.

### Experimental procedures

The participants practiced the BRT movements for 29 days and underwent five measurement days on day 1, day 8, day 15, day 22, and day 29 (Fig. 1A). On each measurement day, five experimental blocks with 3-min rest periods were completed. A block consisted of ten BRT trials and two REP trials, one before and one after the 10 BRT trials (i.e., a total of twelve trials per block) with 12-s resting periods between trials. One trial lasted for 8 s. The start and end of trials were signaled by a sound cue.

On the 24 non-measurement days, the participants were instructed to practice rotating the two balls as fast as possible without seeing their hand or the balls. This practice lasted for 5 min and was conducted at their own home.

### Behavioral measurement and analysis

The movement of digits during the BRT and the REP was recorded with a high-speed video camera (frame rate = 300 Hz, EX-F1, CASIO, JAPAN). Five markers were attached, one on each fingertip, to track fingertip trajectories. The data were analyzed using an open source markerless pose estimation toolbox with deep neural networks (DeepLabCut: http://www.mackenziemathislab.org/deeplabcut)^38,39^. The obtained time-series data underwent a 0.5–5 Hz 2nd-order Butterworth bandpass filter.

We evaluated the time for one cycle during the BRT as an index of performance level (i.e., movement speed). To evaluate the time for one cycle, the time difference between two consecutive maximum extensions of the thumb (i.e., one cycle) was calculated, and then medians of every cycle in each trial (i.e., in each 8 s) were calculated. After that, the median of each block was calculated. Then, the mean value of five blocks on each measurement day were also calculated to compare them across days. We also evaluated the time for one cycle of the REP to check the speed of the reproduced movement using the same procedure as for the BRT.

We evaluated the difference between digit coordination patterns for the BRT and the REP. First, the typical digit coordination pattern of the BRT was calculated in each block for each participant. The relative phases φ between two digits (*f* and *h*) were calculated for each cycle as φ_*hf*_ = *(t*_*f,i*_* − t*_*h,i*_*)/(t*_*f,i*+*1*_* − t*_*f,i*_), where *t*_*h,i*_ and *t*_*f,i*_ indicate the time of the *i*th peak extension of the digits^40^, which has a value between 0–1. Zero means the movements of two digits are completely in phase, and 0.5 means antiphase. Then, for each of the 10 combinations (i.e., thumb-index, thumb-middle, ring-little etc.) of the digits, the median of the relative phase was calculated. Second, we also calculated the medians of the relative phases of the 10 digit combinations for the REP. Third, the differences in the relative phases between the BRT and the REP were calculated for each combination. Then, medians of the difference in each block were calculated for each combination, and then the mean of 10 combinations was evaluated as a difference in digit coordination pattern. Therefore, a smaller difference indicates a greater similarity in movement patterns between BRT and REP. The means of five blocks on each experimental day were also calculated and compared across days. The analysis was conducted by custom-made script in MATLAB (Mathworks, Sherborn, Massachusetts, USA).

### Statistical analysis

The differences in the time spent for one cycle and in the difference in digit coordination pattern across days were tested by a one-way rmANOVA. In addition, the differences in these two indices within day (i.e., across blocks) were also tested by one-way rmANOVA. If the sphericity assumption was violated in Mauchly’s sphericity test, the Greenhouse–Geisser correction coefficient epsilon was used to establish degrees of freedom, and F and P values were recalculated. If significance was observed, post hoc analyses were conducted utilizing paired t-tests with the Bonferroni correction (i.e., significance set at 0.0125). We also compared the normalized values of improvements in the time spent for one cycle and the difference in digit coordination pattern on day 1 using a Wilcoxon signed-rank test ANOVAs were conducted by SPSS, and post-hoc t-tests and the Wilcoxon signed-rank test were performed by MATLAB.

To investigate the relationship between performance level of the BRT and that of the REP, we calculated Pearson's correlation coefficient of the time spent for one cycle in the BRT and the difference in digit coordination pattern. To investigate the relationship between the amount of performance improvement and the recognition capability, we also calculated Pearson's correlation coefficient of the learning rate for the time spent for one cycle in the BRT (i.e., difference between blocks 1 and 5) and the mean difference in digit coordination pattern on day 1 or day 29.

## Data Availability

The datasets analyzed in the current study are available from the corresponding author upon reasonable request.
